# Augmenting care, not replacing it: Generative artificial intelligence and equitable primary care in Southern Africa

**DOI:** 10.4102/safp.v68i1.6340

**Published:** 2026-03-20

**Authors:** Klaus B. von Pressentin, Arun Nair, Mareike Rabe, Marischka de Jong, Ramprakash Kaswa, Indiran Govender

**Affiliations:** 1 Division of Family Medicine, Department of Family, Community and Emergency Care, Faculty of Health Sciences, University of Cape Town, Cape Town, South Africa; 2 Department of Family Medicine, Faculty of Health Sciences, University of the Free State, Bloemfontein, South Africa; 3 Vita Oncology, Cape Town, South Africa; 4 Division of Family Medicine and Primary Care, Stellenbosch University, Cape Town, South Africa; 5 Department of Family Medicine and Primary Health Care, Walter Sisulu University, Mthatha, South Africa; 6 Department of Family Medicine, Mthatha Regional Hospital, Mthatha, South Africa

The experience of primary care among Southern African communities is undermined by unequal access, skilled workforce shortages, fragmented evidence, and poor connectivity. Artificial Intelligence (AI) will not automatically resolve these issues and may worsen disparities. However, when used ethically, AI can uphold core principles such as access, continuity, coordination, comprehensiveness, and person–centredness, as highlighted by Primary Health Care (PHC) frameworks.^1,2^ Strategic frameworks across the African region highlight digital health as a core PHC enabler, provided connectivity, training and system integration are strengthened.^3^ This editorial explores the potential of generative AI (genAI) from the perspectives of patients, providers, and managers. We built on earlier editorials that highlighted both the strategic opportunities of AI for South African primary care and the need for responsible use of conversational AI tools in education and healthcare practice.^4,5^

From the patient’s perspective, conversational AI and automated messaging can enhance initial support and ongoing care, especially when integrated into community platforms. In South Africa’s historically complex sociocultural landscape, trust cannot be assumed. Perceptions of AI and digital literacy vary across settings, with trust tied to transparency and relational integration in clinical encounters. Family medicine and primary care span the intersection of patient–context (beliefs, language, cultural identity, and socioeconomic conditions) and care–context (team, facility, district, and community systems). Uptake depends on a culturally sensitive, ethically grounded approach that centres both the patient–context and the care–context in the co–design and ongoing evaluation of AI–enabled primary care tools. Tools that do not acknowledge relational continuity, cultural nuance, or past experiences of exclusion risk eroding trust rather than enabling it. For example, the AI-driven Ask–A–Question (AAQ) service, integrated into MomConnect, matches user questions with approved responses and escalates urgent cases to humans, thereby increasing access and responsiveness without replacing personal relationships.^6^

At the provider level, the benefits and risks shift from access and communication to workflow, safety and professional identity. GenAI’s most immediate impact may be in reducing the hidden workload that hinders care and contributes to burnout, particularly in tasks such as electronic health documentation, messaging and referral management. While genAI can produce text, summaries and translations, it reshapes consultations from a two-person interaction into a triad that includes AI, requiring new skills and safeguards.^7^ Human review remains essential for trust, safety and accountability in addressing safety concerns, including omissions, distortions and fabricated content, as well as issues of consent, transparency, and accountability. Artificial intelligence literacy, critical appraisal skills and reflective practice will increasingly shape how clinicians learn, reason and communicate. The effective use of AI has significant educational and professional implications across the learning continuum, from students to consultants.

At the system level, AI matters for primary care because it influences how quickly patients can be triaged, escalated, referred and followed up across district health systems. Primary care can benefit from AI tools that streamline service delivery and referrals through rapid decision-making at the point of care. One example is the WHO-endorsed use of computer-aided detection (CAD) for tuberculosis (TB) screening, with several software tools expected to be approved by 2025 for chest X-rays.^8^ However, positive results still require confirmatory testing as evidence from Africa shows variable performance, especially in older adults, those with previous TB, and human immunodeficiency virus (HIV) patients. In many rural and underserved districts, fragile connectivity and uneven digital readiness may limit the safe, equitable deployment of such tools unless infrastructure strengthening proceeds in parallel. Artificial intelligence enhances point–of–care ultrasound (POCUS), improving gestational age estimation and clinician confidence when decision support is available.^9^ Artificial intelligence enables new care models closer to home, such as hospital–at–home and remote monitoring, which reduce length of stay and readmissions and ease strain on the health system. For primary care, the goal is to improve decision-making and strengthen escalation and follow-up pathways, not just add technology for its own sake.

This editorial highlights the interconnected perspectives of patients, providers, and health systems, showing that AI (including genAI) is a socio-technical intervention, and that governance determines whether it advances equity or widens divides. In South Africa, laws such as the *Protection of Personal Information Act (POPIA)* regulate the handling of sensitive health data, while frameworks such as the National Health Normative Standards and the National Digital Health Strategy promote coordinated, person-centred digital health. These governance frameworks act as essential guardrails, ensuring that AI systems are deployed safely, transparently, and in ways that uphold person–centred primary care rather than undermining it.^10^ Realising genAI’s potential and mitigating risks requires ongoing collaboration among primary care providers, innovators, researchers, managers and communities. The 2026 WONCA Statement on Digital Health and AI prioritises human-centred digital transformation in PHC and calls for the formal integration of family doctors into the design and oversight of AI tools to ensure clinical safety, transparency and accountability.^11^ The WONCA has also announced the theme for the 2026 World Family Doctor Day as ‘Compassionate care in a Digital World’.

The South African Academy of Family Physicians (SAAFP) and its *South African Family Practice* journal play a vital role in fostering learning partnerships that extend beyond technology pilots to co-develop programmes addressing urgent system issues. We must accept that AI is here to stay and that its role in PHC will grow, so we should embrace it and learn how to use it effectively. Key questions remain about which competencies are most important, how governance should function within district systems, and how to ensure AI supports human-centred digital care transformation. Addressing these gaps through collaboration is crucial to ensure genAI is shaped not only by algorithms but also by shared decision-making within equitable primary care relationships.
